# Evaluating the performance of interpreting Verbal Autopsy 3.2 model for establishing pulmonary tuberculosis as a cause of death in Ethiopia: a population-based cross-sectional study

**DOI:** 10.1186/1471-2458-12-1039

**Published:** 2012-11-29

**Authors:** Sebsibe Tadesse, Takele Tadesse

**Affiliations:** 1 Institute of Public Health, the University of Gondar, Gondar, Ethiopia

**Keywords:** The InterVA model, Pulmonary tuberculosis, Cause of death

## Abstract

**Background:**

In resource- poor settings, verbal autopsy data are often reviewed by physicians in order to assign the probable cause of death. But in addition to being time and energy consuming, the method is liable to produce inconsistent results. The aim of this study is to evaluate the performance of the InterVA 3.2 model for establishing pulmonary tuberculosis as a cause of death in comparison with physician review of verbal autopsy data.

**Methods:**

A population-based cross-sectional study was conducted from March to April, 2012. All adults aged ≥14 years and died between 01 January 2010 and 15 February 2012 were included in the study. Data were collected by using a pre-tested and modified WHO designed verbal autopsy questionnaire. The verbal autopsy interviews were reviewed by the InterVA model and the physicians. Cohen’s kappa statistic, receiver operating characteristic curves, sensitivity, and specificity values were applied to compare the agreement between the InterVA model and the physician review.

**Results:**

A total of 408 adult deaths were studied. The proportion of tuberculosis-specific mortality was established to be 36.0% and 23.0% by the InterVA model and the physicians, respectively. The InterVA model predicted pulmonary tuberculosis as a cause of death with the probability of 0.80 (95% CI: 0.75-0.85). In classifying all deaths as tuberculosis and non-tuberculosis, the sensitivity and specificity values were 0.82 and 0.78, respectively. A moderate agreement was found between the model and physicians in assigning pulmonary tuberculosis as a cause of deaths [kappa= 0.5; 95% CI: (0.4-0.6)].

**Conclusions:**

This study has revealed that the InterVA model showed a more promising result as a community-level tool for generating pulmonary tuberculosis-specific mortality data from verbal autopsy. The conclusion is believed to provide policymakers with a highly needed piece of information for allocating resources for health intervention.

## Background

Developing countries generally lack consistent, timely, and reliable information on pulmonary tuberculosis (PTB)-specific causes of death (COD) in their populations [1]. Vital registration data are incomplete and contain only few physician-certified deaths [2]. Nevertheless, any meaningful health intervention policy and/or program must be informed by the CODs that are of the greatest importance locally. Verbal autopsy (VA) is a useful tool in such settings to establish the probable COD by interviewing a close caregiver or anyone who can provide witness to the death event [3].

There have been various attempts at validating physician reviews [4,5], but there appears several concerns that arise from using this methodology to interpret VA data. First, physicians may differ systematically in their methods of interpreting VA data owing to their training, experience, and/or perceptions of local epidemiology. Hence, there may be inter- and intra-reviewer variability among physicians that may lead to inconsistencies in COD data hindering reliable temporal and spatial comparisons of mortality [6,7]. Second, the physician review process often demands a considerable amount of physician time and can incur remunerative costs [8].

Various alternative methods to the physician review of VA data have been introduced. These include the use of expert/data-driven algorithms, neural networks, and the InterVA model. Algorithms and neural networks are said to have the advantage of being quicker, more transparent, and more consistent in comparison to the physician review [9-11]. However, investigations on their validity have been so inconclusive that their use remains limited [9,11]. The use of the InterVA model to interpret VA data is a relatively new methodology that has just been explored successfully in a number of settings [12-14]. This computer-based probabilistic program is said to have the advantage of achieving maximum consistency in interpreting VA data [12,14,15]. Moreover, it requires minimal time and labor resources, especially in comparison with the physician review method. Also, it is freely available in the public domain, making it ideal for resource-constrained settings [16].

According to a rural community-based validation study conducted in Butajira, Ethiopia [12], the InterVA model established PTB as a COD for 33% of all deaths. Another study carried out in Kenya [17] showed that 31% of all deaths were due to PTB as assigned by the InterVA model, and only 9.9% as assigned by the physicians. Physicians assigned 6.4% of deaths to PTB while the Model assigned 21.3% to the same cause according to a South African study [18].

Many studies have investigated the validity of the InterVA model as a tool for assigning COD [14,19,20]. A validation study in Kenya [21] indicated the overall diagnostic ability of the model to be 0.82% when compared against the physician. A moderate level of agreement with [kappa=0.42; 95% CI: (0.37-0.48)] was found between the physician and the model in assigning PTB as a COD in a Kenyan comparative validation study [22].

As a means of promoting effective and sustainable TB control and to influence policy decisions, TB mortality information is one of the critical areas for evaluating the progress and impact of interventions. In response to this, the current study is designed to evaluate the performance of the InterVA model as the physician alternative method for generating PTB-specific death data from VAs in northern Ethiopia.

## Methods

**Figure 1:**
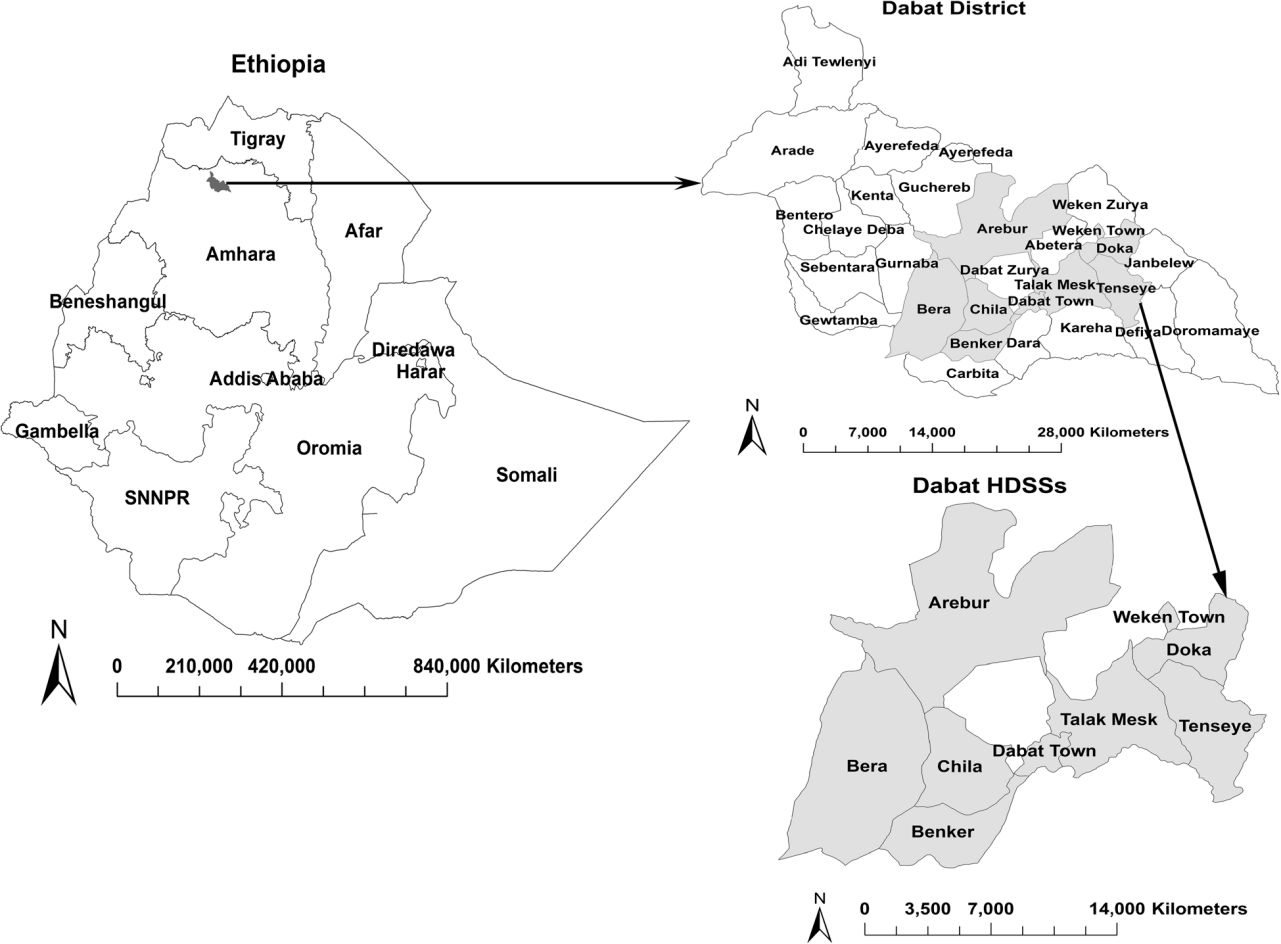
**Map of Ethiopia, Dabat district and Dabat HDSSs**.

A population-based cross-sectional study was implemented from 01 March to 30 April, 2012 in Dabat Health and Demographic Surveillance System site (HDSSs) hosted by the University of Gondar. The site is located in a district known as Dabat, northern Ethiopia (See map, Figure 1), and has an estimated population of 46,165 living in 7 rural and 3 urban "kebeles" (the smallest administrative units in Ethiopia). The local communities largely depend on subsistence agriculture. It has two health centers providing Directly Observed Treatment Short-course (DOTS) for TB cases. Information on vital events, like birth, death, and migration are collected quarterly [23].

### Study participants and data collection

All adults aged ≥14 years and died between 01 January 2010 and 15 February 2012 in the study area were included in the study. The period from 01 January 2010 to 15 February 2012 was preferred in order to obtain an adequate number of deaths without marked implication on recall bias. It is believed that adult deaths are remembered very well.

Pre-tested and modified WHO and INDEPTH [24,25] designed VA questionnaire was used to collect the data. The VA questionnaire included open narrative, medical histories, and closed questions. The narrative section was used to record free explanations of the circumstances of death; the medical history sections were used to extract data from medical certificates, and the closed section dealt with specific signs, symptoms, and conditions leading to death. Three trained supervisors and nine data collectors who had rich experience of field data collection participated in the data collection processes. After obtaining a written informed consent, the data collectors interviewed a close relative, friend, or neighbor of the deceased person who witnessed the death. Considering the usual mourning period in the study area, data were collected after 45 days for recent death events.

The VA questionnaire was translated into Amharic (the local language) and back to English to maintain the consistency of the questions. The training of data collectors and supervisors emphasized issues, such as the selection of eligible respondents, approaching grieving respondents, time of interviews, and compiling narrative responses (ensuring that duration, frequency, severity and sequence of symptoms were mentioned). The principal investigator and the supervisors coordinated the interview process, made spot-checks, and reviewed the completed questionnaires on daily bases to ensure the completeness and consistency of the data collected. They also conducted random quality checks by re-interviewing about 10% of the respondents. The VA questionnaire was pre-tested to identify potential problem areas, unanticipated interpretations, and cultural objections to any of the questions on 25 respondents with similar characteristics with the study subjects nearby Dabat district. Based on the pre-test results, the questionnaire was adjusted contextually. Data entry was carried out by the principal investigator and another independent data clerk and was then compared to check for any variation in results.

### Interpretation of the inter VA model

The InterVA 3.2 Model and the physician reviewed the same basic data from the VA questionnaire independently.

### Physician interpretation

Two independent physicians reviewed each VA questionnaire independently to assign a single COD based on ICD-10. The ICD-10 list had unique codes for diseases, signs, symptoms, abnormal findings, complaints, social circumstances, and external causes of injury [15]. The physicians met subsequently to reach consensus on cases where there were differences of opinion. If no physician consensus was reached after discussion, the COD was regarded as indeterminate. The physicians were trained in procedures on assigning COD and given details of the study area and study population. However, they were not given any special briefing on the probabilistic model so as not to encroach on their professional freedom. In spite of that however, their review process was closely monitored and that they be not direct beneficiaries of the research output was ensured.

### Interpretation of the InterVA model

The model relates a range of input indicators (including age, sex, physical signs and symptoms, medical history, and the circumstances of death) to likely CODs using Bayesian probabilities [15]. The model results in up to three likely causes per case when possible; each associated with a quantified likelihood. To give an estimate of the overall certainty for that patient, the model gives the average likelihood for a maximum of three CODs [16]. In this study, a high prevalence of Malaria and HIV/AIDS were used as basic epidemiological parameters for the model as their prevalence varies from place to place. Data were entered case-by-case into Microsoft visual FoxPro window of the InterVA version 3.2 to assign the possible COD responsible for the death of each individual.

### Comparison of the InterVA model with the physician

The most probable CODs assigned by the model were considered to facilitate comparison with the single CODs which were assigned by the physician. All CODs in both methods were re-categorized into 16 main groups for two reasons. The first reason was to have meaningfully comparable COD categories between both methods. Second, it was more important that the model and the physician arrive at a broad agreement in identifying COD groups with the greatest public health importance at population level, rather than individual-level causes. The list of the 16 main categories used in this study were: maternity-related deaths, PTB, HIV/AIDS, pneumonia, acute/infectious diseases, chronic diseases, malnutrition, homicide, malaria, suicide, transport-related accident, other accidents, digestive diseases, haemoglobinopathy, meningitis, and measles.

If final illness lasted more than 3 weeks, coughing with blood, coughing for more than 3 weeks, excessive night sweating, and weight loss were presented; then the physicians concluded PTB as a leading COD. In cases where they suspected TB-comorbidity, they categorized the COD as a non-TB death in order to increase their level of certainty to establish PTB as a leading COD. Then deaths were aggregated case-by-case to their respective COD categories to determine the cause-specific mortality fractions at community level by using both the InterVA model and the physician review. Receiver operating characteristic (ROC) curve, probability, sensitivity, specificity and Cohen’s kappa statistic with 95% confidence interval (CI) were applied to compare agreement between the InterVA model and the Physician.

In this study, the economic position of the deceased was ranked as poor versus rich based on expenditure–based poverty score [26]. Those who scored below the mean were categorized as poor.

### Ethical considerations

The study protocol was reviewed and approved by the Institutional Ethical Review Board of the University of Gondar. Then, written informed consent was obtained from the study participants who were close relatives, friends, or neighbors of the deceased after explaining the purpose and the procedures of the study. Confidentiality was granted for information collected from each study participant. Study participants found sick at the time of data collection were referred to the nearest health institution for medical treatment. There was no remuneration for family.

## Results

### Characteristics of the study population

A total of 408 VA interviews were successfully completed and reviewed by both the InterVA model and the physician. There was slightly higher proportion of deaths among females, 222 (54.4%). Two hundred eighty-one (68.9%) of the deceased were 50 and above years of age. Most of the deceased, 325 (79.7%) and 298 (90.0%) were married participants and farmers, respectively. As far as education is concerned, 308 (73.0%) of the them were illiterate. With respect to family size, 282(69.1%) deaths occurred among families in which 1–4 people shared the same house. More than two-thirds of all deaths, 277 (67.9%), occurred among economically poor people (Table 1).

**Table 1:** Distribution of all adult deaths by socio-demographic characteristics in Dabat, Ethiopia from 01 January 2010–15 February 2012

**Variables**	**No (n=408)**	**Percent**
Sex		
Female	222	54.4
Male	186	45.6
Age in years		
15-49	127	31.1
50-64	140	34.3
≥65	141	34.6
Marital status		
Single	83	20.3
Married	325	79.7
Educational status		
Illiterate	308	73.0
Literate	100	27.0
Occupational status		
Farmer	298	90.0
Gov’t/Private employee	110	10.0
Family size		
1-4	282	69.1
≥5	126	30.9
Economic status		
Rich	131	32.1
Poor	277	67.9

### Description of PTB-specific mortality rate

Out of the 408 deaths, 329 (80.6%) were successfully assigned a single cause at the first attempt by two physicians. After holding consensus meetings, the physicians successfully assigned a single COD for 61 (15%) more cases. Therefore, on the whole, physicians assigned a single COD for 390 (95.6%) cases. No consensus was reached on 18 (4.4%) cases, which were coded as "indeterminate" by the physicians. Out of these, 5 (1.2%) cases were assigned to be PTB by one of the physician. They established PTB as a COD for 94 (23.0%) of the cases.

The InterVA model assigned PTB as a first COD for 147 (36.0%) cases, as a second COD for 9 (2.2%) cases, and as a third COD for none of the cases. In 10 (2.5%) cases, the InterVA model assigned the COD as "indeterminate". The probabilistic model assigned the likely CODs for all the VAs with a certainty of 75% and standard deviation of 2.8.

In this study, both the InterVA model and the physicians have assigned PTB-specific mortalities for 77 (18.9%) of all deaths in common. Out of these deaths, the respondents correctly predicted PTB as a COD for 52 (67.5%).

The proportion of PTB-specific mortality was found to be higher among illiterate households in which ≥5 persons lived together, farmers, rural dwellers, traditional medicine users, and the economically poor, as shown by the physician review and the InterVA model separately and by both methods at a time. However, there was a slight difference between the two methods (Table 2).

**Table 2:** Distribution of PTB-specific deaths by physicians, by InterVA model, and by both methods in Dabat, Ethiopia from 01 January 2010–15 February 2012

**Variables**	**By physicians**	**By InterVA**	**By both**
	n=94 (%)	n=147 (%)	n=77(%)
Educational Status			
Illiterate	63(67.0)	120(81.6)	52(67.5)
Literate	31(33.0)	27(18.4)	25(32.5)
Family size			
1-4 persons	28(29.8)	58(39.5)	24(31.2)
≥5 persons	66(70.2)	89(60.5)	53(68.8)
Occupational status			
Farmer	73(77.7)	109(74.1)	58(75.3)
Gov’t/private employee	21(22.3)	38(25.9)	19(24.7)
Residence			
Rural	71(75.5)	108(73.5)	57(74.0)
Urban	23(24.5)	39(26.5)	20(26.0)
Health care utilization			
Traditional medicine	57(60.6)	91(61.9)	47(61.0)
Modern medicine	37(39.4)	56(38.1)	30(39.0)
Economic status			
Poor	71(75.5)	118(80.3)	60(77.9)
Rich	23(24.5)	29(19.7)	17(22.1)

### Using the ROC curve to validate the InterVA model for ascertaining PTB as a COD

**Figure 2:**
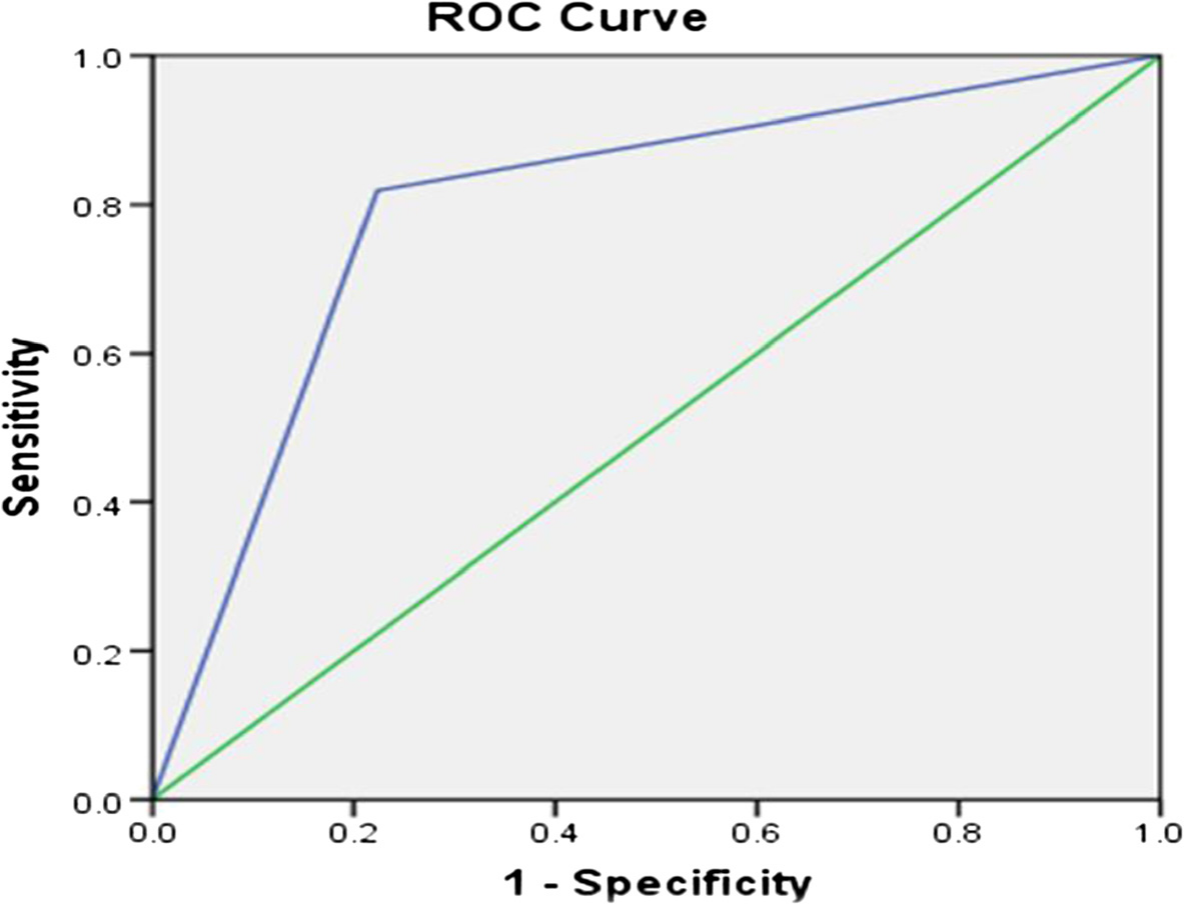
**Comparing the InterVA model with the physician for ascertaining TB as a COD**.

The area under the ROC curve was calculated to measure the overall diagnostic performance (correctly diagnosing all the diseases) of the InterVA model against the physician. For a method to be highly sensitive and specific, the area under the curve should be close to one. The closer the curve follows the left-hand border and the top border of the ROC space, the more accurate the method. The ROC curve has showed that the InterVA model can predict PTB as a COD with the probability (area under the curve) of 0.80 (95% CI: 0.75-0.85) when compared with the physician (Figure 2). The model can estimate PTB as a COD with 81.9% sensitivity and 77.7% specificity. The level of agreement between the model and physician in assigning PTB as a COD was found to be moderate [kappa = 0.5; 95% CI :( 0.4-0.6)].

## Discussion

This study used a probabilistic InterVA model to assess PTB-specific COD in Dabat, northern Ethiopia. The model assigned PTB as a COD for 36.0% of all deaths. This finding was not much far from that of other related studies [12,17]. The diagnostic ability of the model to establish PTB as a COD was evaluated by internally comparing its output with the physician’s review. The model can predict PTB as a COD with the probability of 0.80 (95% CI: 0.75-0.85) when compared with the physician. A similar study [21] has indicated the overall diagnostic ability of the model to be 0.82, indicating a good diagnostic performance of the method. Further studies should be conducted to prove this finding.

A moderate level of agreement was found between the model and the physician in assigning PTB as a COD [kappa = 0.5; 95% CI: (0.4-0.6)]. Almost a similar finding was observed in a Kenyan study [21]. This indicated the temporal and spatial consistency of the InterVA model for establishing PTB as a COD.

Physician review was used as a reference standard to compare the InterVA model. The use of the physician review was the only alternative source of COD assessment for this study population. However, the choice has limitations. The physicians had the advantage of being able to consider detailed information by going through the questionnaire and using their clinical skills and experiences in determining CODs. They might however be influenced by their own biases, particularly for less obvious CODs for which decisions had to be made between equally likely diagnoses. This might have contributed to some of the discordances observed between the two approaches. Another possible limitation of this study could be the cross-sectional study design which might not be appropriate for accurately establishing COD. A longitudinal study design is suggestive. This study used the ROC approach to validate the InterVA model. The ROC methodology assumes comparing something of unknown validity (which here is the InterVA) with something that is 100% correct (which here is the physician). Unfortunately, it is not the case that physicians are 100% correct or consistent in attributing COD. The absence of some variables in the VA questionnaire is a factor challenging the accuracy of the InterVA model. The model does not employ open-ended questions which are more relevant in a society with poor knowledge of symptoms of certain diseases and where more local terms may be used in this case. Another limitation could be the relatively small sample size of the study which might also contribute to the underestimation of the sensitivity and specificity values. Besides, the indeterminate probability of the COD would decrease if more than two physicians reviewed the data. But we couldn’t do this due to the limited budget we had.

## Conclusions

This study has revealed that the InterVA model showed a more promising result as a community-level tool for generating PTB-specific mortality data from verbal autopsy. Further research should be conducted to validate the InterVA model to detect PTB as a COD.

## Competing interests

The authors declare that they have no competing interest.

## Authors’ contribution

Author 1. ST: Initiated the research, wrote the research proposal, conducted the research, did data entry and analysis and wrote the manuscript, Author 2. TT: Involved in the write up of the proposal, the data analysis, and write up of the manuscript. Both authors read and approved the final manuscript.

## Pre-publication history

The pre-publication history for this paper can be accessed here:

http://www.biomedcentral.com/1471-2458/12/1039/prepub
